# c-MYC responds to glucose deprivation in a cell-type-dependent manner

**DOI:** 10.1038/cddiscovery.2015.57

**Published:** 2015-11-23

**Authors:** S Wu, X Yin, X Fang, J Zheng, L Li, X Liu, L Chu

**Affiliations:** 1 State Key Laboratory of Cell Biology, Institute of Biochemistry and Cell Biology, Shanghai Institutes for Biological Sciences, Chinese Academy of Sciences, 320 Yue-Yang Road, Shanghai 200031, China; 2 Jiangsu Center for the Collaboration and Innovation of Cancer Biotherapy, Cancer Institute, Xuzhou Medical College, Xuzhou, Jiangsu 221002, China; 3 Center of Clinical Oncology, Affiliated Hospital of Xuzhou Medical College, Xuzhou, Jiangsu 221002, China

## Abstract

Metabolic reprogramming supports cancer cells’ demands for rapid proliferation and growth. Previous work shows that oncogenes, such as MYC, hypoxia-inducible factor 1 (HIF1), have a central role in driving metabolic reprogramming. A lot of metabolic enzymes, which are deregulated in most cancer cells, are the targets of these oncogenes. However, whether metabolic change affects these oncogenes is still unclear. Here we show that glucose deprivation (GD) affects c-MYC protein levels in a cell-type-dependent manner regardless of P53 mutation status. GD dephosphorylates and then decreases c-MYC protein stability through PI3K signaling pathway in HeLa cells, but not in MDA-MB-231 cells. Role of c-MYC in sensitivity of GD also varies with cell types. c-MYC-mediated glutamine metabolism partially improves the sensitivity of GD in MDA-MB-231 cells. Our results reveal that the heterogeneity of cancer cells in response to metabolic stress should be considered in metabolic therapy for cancer.

## Introduction

Proliferating cells and most cancer cells produce energy and macromolecules through an unusual metabolic pathway compared with non-proliferating or differentiated cells. They metabolize glucose from oxidative phosphorylation to glycolysis regardless of the availability of oxygen, and this phenomenon is known as aerobic glycolysis or Warburg effect.^1^ Comparing with oxidative phosphorylation, glycolysis is a less efficient-way to consume glucose, at least in term of ATP production. One explanation is that a lot of intermediates are produced by glycolysis to meet the bioenergetic and biosynthetic demands of rapid proliferation.^2^ In addition, reduction of the demand of oxygen helps cancer cells survive in low-oxygen condition.^3,4^

A series of enzymes involved in glucose metabolism are responsible for the metabolic alterations during tumorigenesis, for example, glucose transporter 1 (GLUT1),^5^ phosphofructokinase (PFK),^6^ phosphoglycerate kinase 1 (PGK1),^7^ pyruvate kinase, muscle (PKM),^8^ lactate dehydrogenase A (LDHA).^9^ These genes are deregulated in most cancer cells. Most proliferating cancer cells highly express M2 isoform of pyruvate kinase M (PKM2) instead of PKM1 in normal differentiated cells.^10^ It is believed that low catalytic activity of PKM2 allows accumulation of glycolytic intermediates for macromolecular biosynthesis to increase cell proliferation and tumor growth.^11,12^ Phosphofructokinase/fructose-2,6-bisphosphatase B3 gene (PFKFB3) is more selectively expressed in human cancers than other splice variants.^13^ PFKFB3 catalyzes a rate-limiting step of glycolysis with high kinase activity, resulting in promotion of glucose consumption and glycolytic flux.^14^ LDHA promotes glycolysis and tumor cell growth by regulating the intracellular NADH/NAD^+^ redox homeostasis.^15,16^ Excretion of lactate to extracellular matrix changes the microenvironment and promotes tumor migration and invasion.^17^

Deregulation of oncogenes, tumor suppressors or related signaling pathways drives the metabolic changes. A large amount of metabolic enzymes are regulated by oncogene c-MYC, HIF1α and KRAS, tumor suppressor gene P53 or PI3K/AKT^18^ and AMPK signaling pathways.^19^ For instance, c-MYC not only regulates expression of hexokinase 1 (HK1), PFK, PDK1 and LDHA,^19^ but also promotes mitochondrial gene expression and mitochondrial biogenesis.^20^ Gao *et.al*. reported that c-MYC promotes glutamine metabolism by enhancing the expression of glutaminase (GLS).^21^ Glutamine is important for cell proliferation as a nitrogen donor and carbon source in glucose-independent way.^22^ P53 expression also controls a large number of metabolic genes including GLUT1,^23^ TP53-induced glycolysis and apoptosis regulator (TIGAR),^24^ synthesis of cytochrome C oxidase 2 (SCO2).^25^

However, cancer cell metabolism reprogramming is not only the result of deregulation of metabolic enzymes, oncogenes or tumor suppressor genes. Metabolic alteration might be an actively adaptive process. Glycolysis provides advantages for cancer cells to survive in the microenvironment with low oxygen and nutrient.^26^ Metabolic changes are not only the downstream of oncogenic pathways, but also an upstream event that regulates the signaling pathways. Numerous studies have shown that PI3K/AKT,^27,28^ AMPK^29,30^ and mTOR signaling pathways^31,32^ are activated upon glucose deprivation. In addition, glucose controls the intracellular acetylation level of proteins, including mitochondrial proteins,^33^ metabolic enzymes^11,34^ and histones.^35^

In the present study, we assessed the effect of glucose deprivation (GD) on c-MYC protein in several cancer cell lines. We found that GD affected c-MYC protein levels in a cell-type-dependent manner. GD increased c-MYC transcription in both HeLa cells and MDA-MB-231 cells, but decreased c-MYC protein stability only in HeLa cells. PI3K and SIRT1 were involved in the regulation of c-MYC protein stability in HeLa cells. In addition, we showed that c-MYC played different role in the sensitivity to GD in HeLa cells and MDA-MB-231 cells. Resistance of GD in MDA-MB-231 cells was partially associated with c-MYC-mediated glutamine metabolism. Our results provide evidence about the heterogeneity of cancer cells to GD and suggest that targeting glucose metabolism for cancer therapy should be discriminately evaluated.

## Results

### Glucose deprivation affects c-MYC protein levels in a cell-type-dependent manner

**Figure 1:**
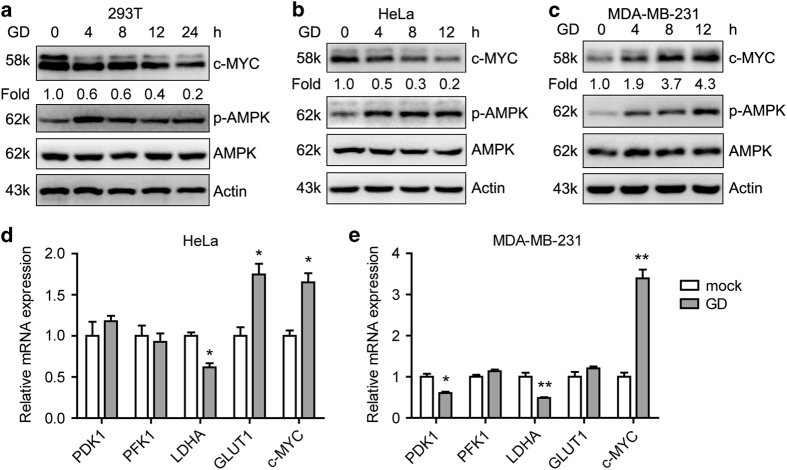
Glucose deprivation differentially affects c-MYC protein levels in different cells. (**a–c**) Western blot detection of c-MYC levels in HEK293T (**a**), HeLa (**b**) and MDA-MB-231 (**c**) cells exposed to GD (0.5 mM) for the indicated intervals. (**d** and **e**) Quantitative RT-PCR detection of PDK1, PFK1, LDHA, GLUT1 and c-MYC levels in HeLa (**d**) and MDA-MB-231 (**e**) cells under GD condition for 12 h. Values represent the relative induction normalized to 18S. *P<0.05, **P<0.01 *versus* mock. Data of three independent experiments are shown.

Previous work showed that oncogenes, such as c-MYC, drive the metabolic reprogramming.^36^ To determine whether metabolic change affects c-MYC, different cell lines were subjected to glucose deprivation (GD). Phosphorylation of AMPK was used as an indicator of the effect of GD. GD decreased c-MYC protein levels in HEK293T and HeLa cells, but increased c-MYC protein levels in MDA-MB-231 cells (Figures 1a–c). Intriguingly, GD elevated c-MYC mRNA levels in HeLa cells and to a great extent in MDA-MB-231 cells (Figures 1d and e). PDK1, LDHA and GLUT1 were also changed in response to GD. These data provide evidence of the mutual regulation between glucose metabolism and the metabolic-related genes.

### Glucose deprivation decreases c-MYC protein stability in HeLa cells but not in MDA-MB-231 cells

**Figure 2:**
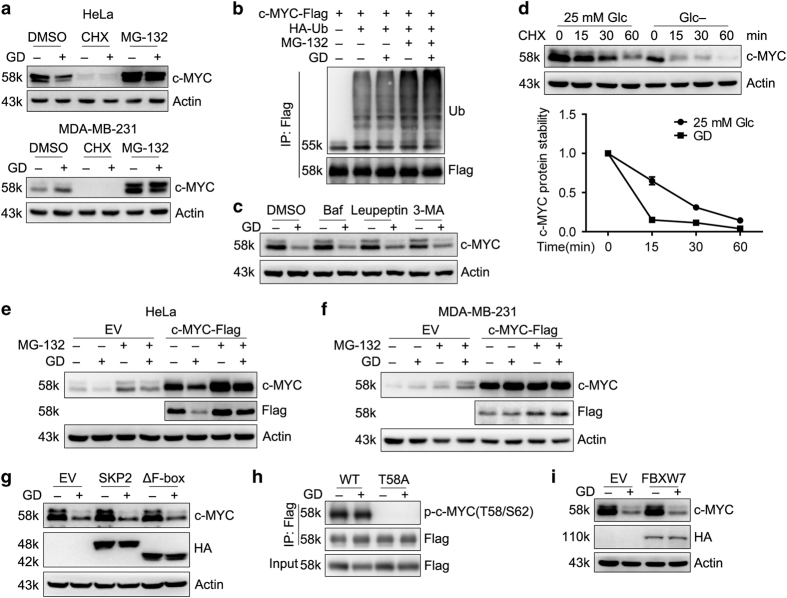
Glucose deprivation differentially affects c-MYC protein stability in HeLa and MDA-MB-231 cells. (**a**) Western blot detection of c-MYC in HeLa and MDA-MB-231 cells treated with CHX (0.1 mM) and MG-132 (10 *μ*M) in the presence or absence of 25 mM glucose for 12 h. (**b**) HEK293T cells cotransfected with c-MYC-Flag and HA-Ub were treated with MG-132 in the presence or absence of glucose for 12 h. Ubiquitination of c-MYC was determined. (**c**) Western blot detection of c-MYC in HeLa cells treated with Bafilomycin A1 (Baf) (500 nM), Leupeptin (10 *μ*g/ml) or 3-MA (1 mM) in the presence or absence of glucose for 12 h. (**d**) HeLa cells were treated with CHX for the indicated time in the presence or absence of glucose. Representative immunoblots of c-MYC in three independent experiments were shown. Bottom, quantification of the c-MYC levels was shown. (**e** and **f**) HeLa (**e**) and MDA-MB-231 (**f**) stable cells ectopically expressing c-MYC-Flag were treated with MG-132 in the presence or absence of glucose. Exogenous c-MYC stability was examined. (**g**) Western blot detection of c-MYC in HeLa stable cells expressing WT HA-SKP2 or HA-tagged dominant negative mutant HA-SKP2(ΔF-box) under GD condition for 12 h. (**h**) HEK293T cells were transfected with WT-c-MYC-Flag or c-MYC(T58A)-Flag. Cell lysates were subjected to immunoprecipitation and phosphorylation of c-MYC was examined. (**i**) Western blot detection of c-MYC in HeLa stable cells expressing FBXW7 treated as in **g**.

We first investigated why c-MYC protein levels were decreased even when the mRNA levels were elevated in response to GD in HeLa cells. HeLa and MDA-MB-231 cells were treated with protein synthesis inhibitor cycloheximide (CHX) or proteasomal inhibitor MG-132, respectively. The half-life of c-MYC is short and 12-h treatment of CHX completely depleted c-MYC protein in both HeLa and MDA-MB-231 cells. On the contrary, MG-132 significantly induced accumulation of c-MYC in both cells and blocked GD-mediated decrease of c-MYC in HeLa cells (Figure 2a). GD also increased the ubiquitination of c-MYC in the presence of MG-132 (Figure 2b). We used lysosomal protease inhibitors bafilomycin A1, Leupeptin and 3-MA to exclude the possibility that c-MYC was degraded through autophagy in HeLa cells under GD condition (Figure 2c). CHX chase experiment indicated that the half-life of c-MYC in HeLa cells was decreased in the absence of glucose (Figure 2d).

To evaluate the effect of GD on c-MYC protein stability, cells ectopically expressing c-MYC were treated with MG-132 under GD condition. Comparing with HeLa cells, exogenous c-MYC in MDA-MB-231 cells was not affected after GD treatment (Figures 2e and f), indicating that GD does not affect c-MYC protein stability in MDA-MB-231 cells. In addition, ectopically expressing c-MYC driven by CMV promoter also excluded the possibility that c-MYC was controlled by IRES-dependent translation.^37^ These data explain that c-MYC protein levels were accumulated in MDA-MB-231 cells but not in HeLa cells in response to GD.

SKP2 and FBXW7 are two main E3 ubiquitin ligases of c-MYC.^38,39^ However, overexpression of SKP2 or an F-box deleted dominant negative mutant SKP2(ΔF-box)^40^ did not affect GD-mediated degradation of c-MYC in HeLa cells (Figure 2g). c-MYC is degraded by FBXW7 in a phosphorylation-dependent manner.^41^ Phosphorylation of Thr58 and Ser62 was not affected in response to GD (Figure 2h) and overexpression of FBXW7 also had no effect on c-MYC protein regardless of glucose (Figure 2i). This implies that other E3 ubiquitin ligase may be responsible for GD-induced degradation of c-MYC.

### Cell-type-dependent response of c-MYC to GD is not dependent on P53

To figure out how c-MYC protein stability vary with cell types in response to GD, c-MYC protein expressions were examined in several cell lines (Table 1). TP53, Ras and PIK3CA have important role in glucose metabolism,^12,42,43^ and are commonly mutated in cancers.^44–47^ We summarized the mutations in these cell lines by referring to Catalogue of Somatic Mutation in Cancer (COSMIC), and noticed that c-MYC protein decreased in four cell lines with wild-type P53 (HEK293T, HeLa, MCF7, HCT116), and increased in majority cell lines with mutated P53 or P53-null after GD treatment (Table 1).

**Figure 3:**
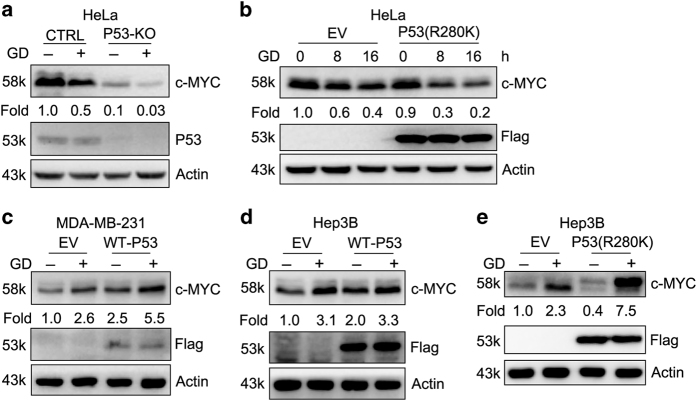
Cell-type-dependent response of c-MYC to GD is not caused by p53 mutation. (**a**) Western blot detection of c-MYC in HeLa control or P53 KO cells under GD condition for 12 h. (**b**) Western blot detection of c-MYC in HeLa stable cells expressing P53(R280K) under GD condition for the indicated intervals. (**c–e**) Western blot detection of c-MYC in MDA-MB-231 stable cells expressing WT-P53 (**c**), Hep3B stable cells expressing WT-P53 (**d**) or P53(R280K) (**e**) under GD condition for 12 h.

P53 was reported functionally inactive in HeLa cells because of expression of human papiloma virus E6.^48^ To detect the role of P53 in GD-mediated degradation of c-MYC, we knocked out P53 in HeLa cell line using CRISPR/Cas9 system. c-MYC protein levels in P53 KO cells were significantly decreased compared with control cells, and were further declined under GD condition (Figure 3a). Overexpression of mutant P53(R280K) in HeLa cells did not reverse the decreased expression of c-MYC caused by GD (Figure 3b). In addition, c-MYC protein levels were still upregulated after GD in WT-P53 overexpression MDA-MB-231 cells (Figure 3c). Ectopically expressing of WT-P53 or mutant P53(R280K) in the P53-null Hep3B cell line did not reverse the increased expression of c-MYC caused by GD (Figures 3d and e). These data indicate that cell-type-dependent different response of c-MYC to GD is not caused by P53 mutation.

### Glucose deprivation increases c-MYC transcription partially through ERK signaling pathway

**Figure 4:**
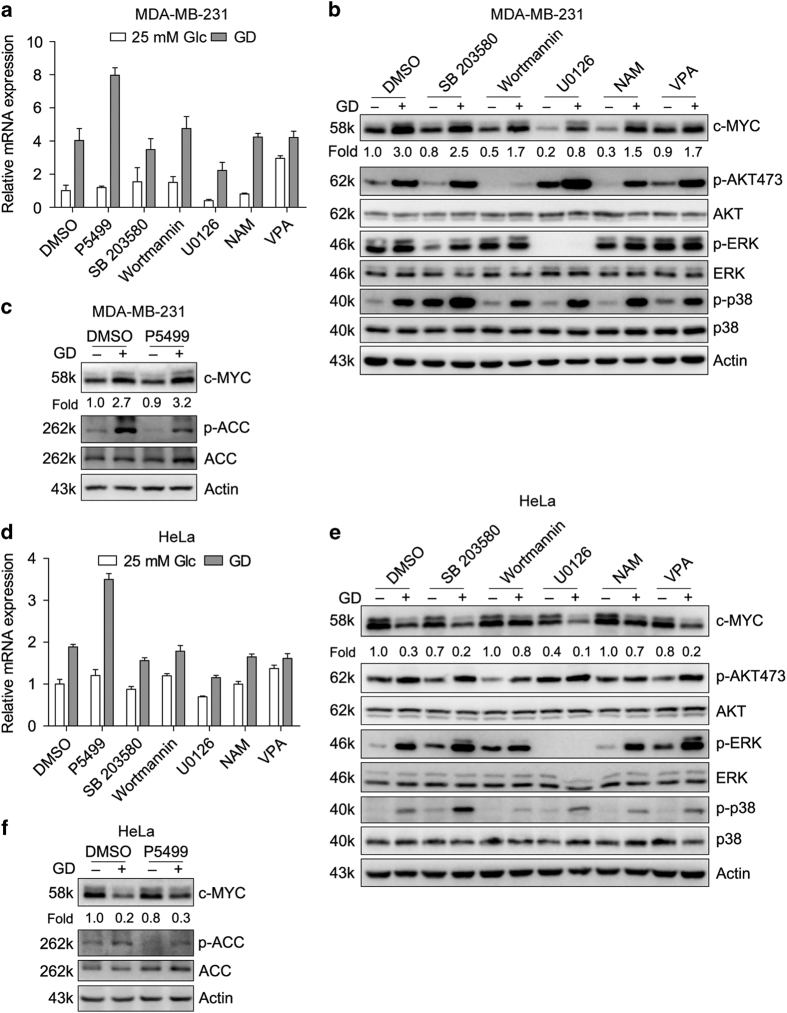
Glucose deprivation increases c-MYC transcription partially through ERK signaling pathway in MDA-MB-231 cells. Quantitative RT-PCR (**a**) and Western blot (**b** and **c**) detection of c-MYC in MDA-MB-231 cells treated with different chemical inhibitors for 12 h in the medium with or without glucose. The indicated chemical inhibitors are AMPK inhibitor P5499 (10 *μ*M), p38/MAPK inhibitor SB 203580 (10 *μ*M), PI3K/AKT inhibitor Wortmannin (10 *μ*M), ERK/MEK inhibitor U0126 (10 *μ*M), SIRT inhibitor NAM (1 mM) and HDAC inhibitor VPA (1 mM). (**d–f**) HeLa cells were treated and detected as that of MDA-MB-231 in **a–c**. Quantitative RT-PCR values were relative to the DMSO group with 25 mM Glc and normalized to 18S. Data of three independent experiments are shown.

Figures 1d and e showed that c-MYC transcription was elevated in both HeLa and MDA-MB-231 cells. Given that GD affects several signaling pathways such as p38/MAPK,^49^ PI3K/AKT,^50^ ERK^51^ and AMPK,^52^ chemical inhibitors were applied to examine which pathway is involved in GD-mediated elevation of c-MYC transcription. Phosphorylation specific antibodies p-p38, p-AKT, p-ERK and p-ACC were used as indicators to show the effects of the inhibitors. We observed that p38/MAPK, PI3K/AKT, ERK and AMPK pathways were all activated after GD and only ERK inhibitor U0126 blocked both mRNA and protein levels in HeLa and MDA-MB-231 cells whether glucose existed or not (Figure 4). However, c-MYC mRNA and protein levels were still elevated in MDA-MB-231 cells and reduced in HeLa cells after GD when ERK signaling was inhibited by U0126. These data indicate that ERK pathway is partially involved in GD-mediated c-MYC transcription activation in both HeLa and MDA-MB-231 cells.

### PI3K, but not AKT, is involved in GD-mediated decrease of c-MYC protein stability in HeLa cells

**Figure 5:**
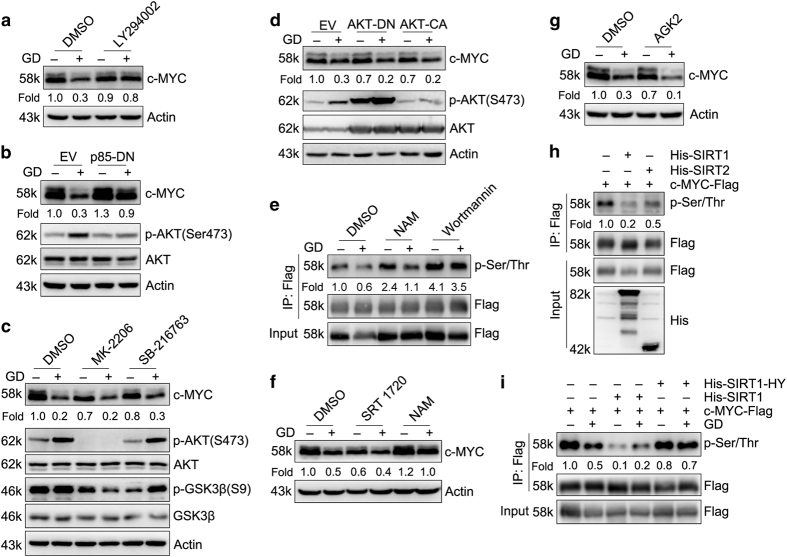
Both SIRT1 and PI3K regulates c-MYC phosphorylation and the following protein stability under GD condition. (**a**) Western blot detection of c-MYC in HeLa cells treated with LY294002 (10 *μ*M) under GD condition for 12 h. (**b**) Western blot detection of c-MYC in HeLa stable cells expressing dominant negative mutant p85-DN under GD condition for 12 h. (**c**) Western blot detection of c-MYC in HeLa cells treated with MK-2206 (10 *μ*M) or SB-216763 (10 *μ*M) under GD condition for 12 h. (**d**) Western blot detection of c-MYC in HeLa stable cells expressing constitutively active mutant AKT-CA or dominant negative mutant AKT-DN under GD condition for 12 h. (**e**) Effects of NAM and Wortmannin on phosphorylated Ser/Thr of c-MYC immunoprecipitated from HEK293T cell lysates under GD condition. (**f** and **g**) Western blot detection of c-MYC in HeLa cells treated with SRT1720 (10 *μ*M) (**f**) or AGK2 (10 *μ*M) (**g**) under GD condition for 12 h. NAM was used as a positive control. (**h**) MYC-Flag was cotransfected with His-SIRT1 or His-SIRT2 into HEK293T cells. Phosphorylated Ser/Thr of immunoprecipitated c-MYC was examined. (**i**) MYC-Flag was cotransfected with His-SIRT1 or kinase-dead His-SIRT1-HY into HEK293T cells and maintained under 25 mM glucose or GD condition for 12 h. Phosphorylated Ser/Thr of immunoprecipitated c-MYC was examined.

Meanwhile, we found that degradation of c-MYC under GD condition in HeLa cells were prevented by PI3K inhibitor Wortmannin and SIRT inhibitor NAM (Figure 4e). Another PI3K inhibitor LY294002 showed similar effect as Wortmannin (Figure 5a). PI3Ks are composed of a catalytic subunit (p110) and a regulatory subunit (p85).^53^ To specifically confirm the role of PI3Ks in GD-mediated degradation of c-MYC in HeLa cells, we constructed a dominant negative mutant of p85 (p85-DN), which lose the regulatory activity of catalytic subunit p110.^54^ p85-DN prevented GD-mediated degradation of c-MYC protein in consistent with the effect of Wortmannin and LY294002 (Figure 5b).

AKT is the main target of PI3K, and is activated by growth factor in a PI3K-dependent way.^55^ Activated AKT phosphorylates and inactivates glycogen synthase kinase 3 (GSK3), and then prevents GSK3*β*-mediated phosphorylation and degradation of downstream targets such as c-MYC^56^ and cyclin D1.^57^ We found that both AKT inhibitor MK-2206 and GSK3*β* inhibitor SB-216763 had no significant effect on GD-mediated degradation of c-MYC (Figure 5c). Inhibition of AKT by a dominant negative mutant AKT-DN or activation of AKT by a constitutively active mutant AKT-CA^58^ had no distinct effect on c-MYC protein levels as similar as p85-DN (Figure 5d). These results demonstrate that GD induces c-MYC degradation through a PI3K-, but not AKT-, dependent way.

### Both PI3K and SIRT1 regulate c-MYC phosphorylation and the following protein stability under GD condition

The above data showed that Wortmannin and NAM abolished GD-mediated degradation of c-MYC. To investigate how PI3K and SIRT affect c-MYC protein stability, we examined the phosphorylation of c-MYC treated with NAM or Wortmannin under GD condition. Results showed that GD decreased c-MYC phosphorylation. Both inhibitors, especially Wortmannin, significantly blocked the GD-mediated dephosphorylation of c-MYC (Figure 5e). Considering that NAM is a SIRTs inhibitor, we supposed that the effect of NAM on c-MYC phosphorylation is indirect.

We further found that SIRT1 activator SRT1720 could mimic the effect of GD on c-MYC protein levels (Figure 5f). However, SIRT2 specific inhibitor AGK2 failed to block GD-mediated degradation of c-MYC protein (Figure 5g). This indicates that SIRT1 is involved in GD-mediated degradation of c-MYC in HeLa cells.

To further confirm the function of SIRT1, we cotransfected SIRT1 or SIRT2 with c-MYC. SIRT1 effectively decreased c-MYC phosphorylation and protein level, whereas SIRT2 showed only minor effect on c-MYC phosphorylation (Figure 5h). In addition, GD-induced dephosphorylation of c-MYC was markedly enhanced by overexpression of SIRT1 and inhibited by kinase-dead mutant SIRT1-HY (Figure 5i).^59^ Collectively, these data indicate that both PI3K and SIRT1 regulate GD-mediated dephosphorylation and degradation of c-MYC.

### c-MYC-mediated glutamine metabolism is involved in the resistance to GD in MDA-MB-231 cells

**Figure 6:**
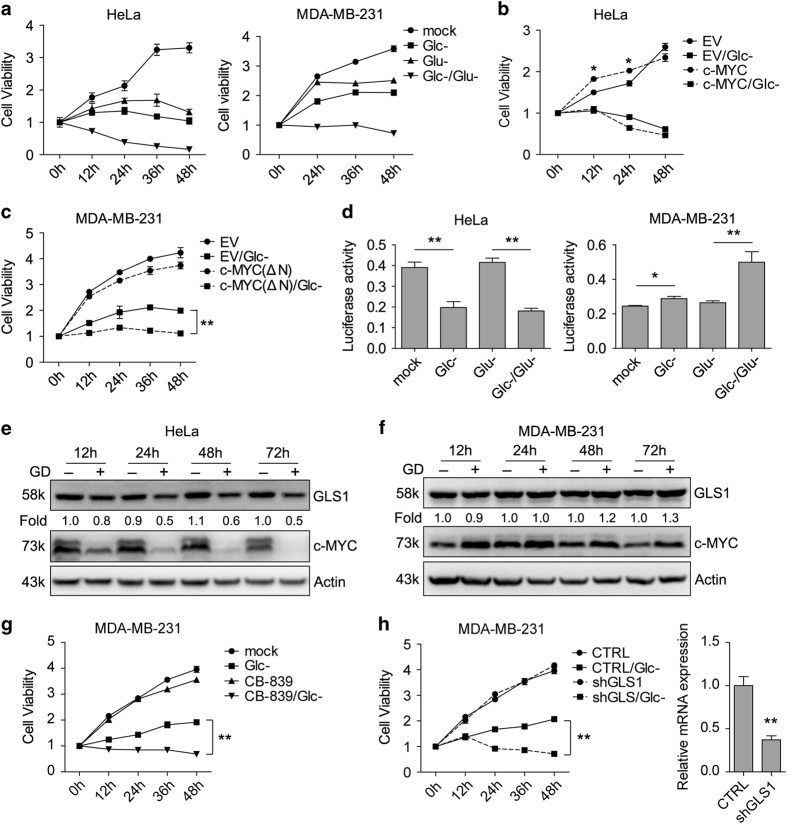
c-MYC-mediated glutamine metabolism is involved in the resistance to GD in MDA-MB-231 cells. (**a**) HeLa and MDA-MB-231 cells were maintained under the indicated medium for different intervals. Cell viabilities were examined by MTT assay. (**b**) HeLa stable cells expressing MYC were maintained under GD condition for different intervals and cell viabilities were examined by MTT. (**c**) MDA-MB-231 stable cells expressing c-MYC(ΔN) were treated as in **b**. (**d**) GLS1-3′-UTR was transfected into HeLa or MDA-MB-231 cells and maintained under the indicated medium for 36 h. Luciferase activities were examined. *P<0.05, **P<0.01. (**e** and **f**) Western blot detection of GLS1 and c-MYC in HeLa (**e**) and MDA-MB-231 (**f**) cells under GD condition for different intervals. (**g**) MDA-MB-231 cells were treated with CB-839 (10 *μ*M) under GD condition for different intervals and cell viabilities were examined by MTT. (**h**) MDA-MB-231 stable cells infected with lenti-shGLS1 were treated as in (**b**). (Right) Examination of GLS1 knockdown efficiency by quantitative RT-PCR. Values are normalized to 18S and relative to control group. MTT in (**a**–**c**, **g** and **h**) were repeated three times, and values are shown as fold changes *versus* 0 h group.

c-MYC has been reported to affect cell viability under GD condition^60,61^ and this effect might vary with cell type.^62^ We found that HeLa and MDA-MB-231 cells showed distinct response to glucose or glutamine deprivation (Figure 6a). Overexpression of c-MYC in HeLa cells increased cell viability in normal medium, but had negligible effect on cell viability under GD condition (Figure 6b). By contrast, overexpression of a transactivation domain-deleted mutant MYC(ΔN)^63^ markedly sensitized MDA-MB-231 cells to GD (Figure 6c). These findings suggest that c-MYC might exert different role in HeLa and MDA-MB-231 cells under GD condition.

Given that c-MYC enhances glutamine metabolism reprogramming,^64,65^ we speculate that c-MYC promotes glutamine metabolism in MDA-MB-231 cell. To test this hypothesis, we performed luciferase assay to examine the effect of GD on the 3′-UTR reporter activity of GLS1, which is the target of c-MYC and responsible for glutamine metabolism.^21^ Luciferase assay and immunoblot showed that GD decreased GLS1 3′-UTR activity and protein levels in HeLa cells but displayed an opposite effect in MDA-MB-231 cells (Figures 6d–f). In addition, both GLS1 inhibitor CB-839 (Figure 6g) and shRNA interference of GLS1 (Figure 6h) sensitized MDA-MB-231 cells to GD. Collectively, these results demonstrate that resistance to GD in MDA-MB-231 cells is partially due to glutamine metabolism driven by c-MYC.

## Discussion

Cancer cells need large amount of energy and intermediates to fuel their rapid proliferation and growth. To meet the demand, cancer cells experience metabolic reprogramming. This change is thought to result from deregulation of oncogenes or tumor suppressor genes.^36,66,67^ In this study, we showed that GD could alter oncogene c-MYC levels in different cancer cell lines. Our present data unveil a mutual regulation between metabolism and oncogenes or tumor suppressor genes.

The extremely different background of each cell line makes it difficult to identify which molecule determines the change of c-MYC under GD condition. A feasible way is to expand the number of cell lines for detection. We examined c-MYC expression in 17 cell lines and analyzed some key point mutations related to glucose metabolism. It seems that there is a close correlation between P53 mutation and c-MYC change in response to GD (Table 1). However, our data indicated that P53 cannot determine the different response of c-MYC to GD in HeLa (WT P53), MDA-MB-231(mutant P53) and Hep3B (P53 null) cells (Figure 3). So far, we cannot confirm that P53 is responsible for GD-mediated variant c-MYC change in other cell lines. Further studies are required to clarify the molecular regulators.

We found that GD increased c-MYC transcription levels partially through ERK signaling pathway (Figure 4). This is consistent with previous data.^68^ Besides ERK, Blackburn *et al.* reported that JNK1 is also involved in GD-induced increase of c-MYC transcription. In any way, c-MYC transcription in both HeLa and MDA-MB-231 cells are elevated in response to GD. Here our study focused on the different response of c-MYC under GD condition between HeLa and MDA-MB-231 cells. This is the reason that we pay much attention to c-MYC protein levels other than transcription levels. Although we demonstrate that PI3K and SIRT1 could block GD-mediated degradation of c-MYC, the effect may not be direct, because PIK3CA localizes in the cytoplasm and membrane, while c-MYC is mainly accumulated in the nucleus. SIRT1 might affect c-MYC phosphorylation and degradation through deacetylation.

GD differentially affects c-MYC protein stability in HeLa and MDA-MB-231 cells (Figures 2e and f). There are three possibilities. First, mutation of c-MYC inhibits its degradation. Second, specific E3 ubiquitin ligase, which is required for c-MYC degradation, loses its function. Third, post-translational modifications, especially phosphorylation,^41^ have important roles in c-MYC proteins stability.^69^ Deregulation of post-translational modification may inhibit c-MYC following degradation. We noticed that c-MYC is not mutated in HeLa and MDA-MB-231 cells and the known c-MYC E3 ubiquitin ligases, such as SKP2 and FBXW7, are normal in both cells. So the most possible reason is that there are other different factors between HeLa and MDA-MB-231 cells that affect the phosphorylation of c-MYC.

Several factors are reported to be responsible for the sensitivity to GD in different cancer cells.^68,70,71^ We found that c-MYC-mediated glutamine metabolism enhances the tolerance to GD in MDA-MB-231, but not in HeLa cells (Figure 6). This is consistent with previous data that glucose metabolism is heterogeneous in different cells.^62,72^ Our data provide evidence to show the role of c-MYC in the transition from glucose metabolism to glutamine metabolism under GD condition.

Targeting cancer cell metabolism is a promising way for cancer therapy. An in-depth understanding of metabolic regulation in cancer cells will provide new insight for effective therapies. Our results suggest that the heterogeneity should be considered in metabolic cancer therapy targeting glucose metabolism. Change of c-MYC might be thought as an indicator of choosing targeting glucose metabolism or a combination of targeting glucose and glutamine metabolism.

## Materials and Methods

### Cell culture and reagents

HeLa cell was cultured with DMEM (Invitrogen, CA, USA) supplemented with 10% fetal bovine serum (Biochrom, Berlin, Germany). MDA-MB-231 cell was cultured with Leibovitz’s L-15 (Invitrogen) supplemented with 10% fetal bovine serum. All the other cells were maintained following the ATCC guideline.

DMEM (no glucose), DMEM (no glutamine) and DMEM (no glucose, no glutamine) were purchased from Life Technologies (Carlsbad, CA, USA). Anti-c-MYC antibody was purchased from Abcam (Cambridge, MA, USA). Anti-GLUT1 (H-43), Ub (P4D1) and P53 (Pab 1801) antibodies were purchased from Santa Cruz Biotechnology (Dallas, TX, USA). Anti-Flag, His, HA and Actin antibodies were purchased from Abmart (Shanghai, China). Anti-p-AKT (Ser473), AKT, p-GSK3*β* (Ser9), GSK3*β*, p-AMPK(Thr172), AMPK, p-ACC(Ser79), ACC, p-p38(Thr180/Tyr182), p38, p-Ser/Thr, p-ERK1/2(Thr202/Tyr204) and ERK antibodies were purchased from Cell Signaling Technology (Danvers, MA, USA). Anti-c-MYC(p-Thr58/Ser62) antibody was purchased from GeneTex (Irvine, CA, USA). Anti-GLS antibody was purchased from ABGENT (San Diego, CA, USA).

Chemical inhibitors: cycloheximide, bafilomycin A1, 3-MA and SB-216763 were purchased from Sigma (St. Louis, MO, USA). Leupeptin was purchased from Amresco (Solon, OH, USA). MG-132, Wortmannin, P5499, MK-2206, SRT1720, AGK2 and CB-839 were purchased from Selleck (Houston, TX, USA). VPA was purchased from Millipore (Billerica, MA, USA). SB 203580, U0126 and NAM were purchased from Beyotime (Jiangsu, China).

### Lentiviral package and infection

pLVX system was used for lentivirus package. HEK293T cell was transfected by calcium phosphate according to the standard protocol. For stable transfection, cells were infected with lentiviruses and were screened by incubation with 1–2 *μ*g/ml puromycin beginning at 48 h post-infection.

### Luciferase reporter assay

GLS 3′-UTR was cloned from HeLa cDNA and inserted into pmirGLO. Luciferase plasmids were transfected into HeLa cell or MDA-MB-231 cell using Lipofectamine 2000 (Invitrogen). Cells were cultured with DMEM (no glucose) 24-h post-transfection. Luciferase assay was performed using the Dual Luciferase Assay System (Promega, Madison, WI, USA) after treatment for 36 h. Relative luciferase activity was shown as the ratio to Renilla luciferase activity.

### Western blot and immunoprecipitation

Cells were collected with IP lysis buffer (Beyotime, Jiangsu, China) and centrifuged at 12 000 r.p.m. for 10 min at 4 °C. The supernatant was incubated with appropriate antibodies in addition to protein G beads (Roche, Basel, Switzerland) overnight at 4 °C. Immunoprecipitates were washed five times with PBS and resuspended in 60 *μ*l SDS loading buffer. Samples were separated by 10% SDS-PAGE and transferred to PVDF membrane (Millipore). Membranes were blocked with 5% non-fat milk or 5% BSA and then incubated with primary antibodies and secondary antibodies. Signals were visualized by chemiluminescence. Quantitative analysis was performed using Gelpro software.

### RT-PCR and quantitative PCR

Total RNAs were isolated using the TRIzol reagent (Invitrogen) according to the manufacturer’s instruction. Extracted RNAs were used for RT-PCR with the ReverTra Ace qPCR RT kit (TOYOBO, Osaka, Japan). Quantitative PCR was performed using Bio-Rad CFX96 Real-Time PCR Systems. Changes in gene expression were normalized to 18S. The primers were as follow: PDK1-F: 5′-TTACGCACAATACTTCCA-3′ and PDK1-R: 5′-AGAGCCTTAATGTAGATAACT-3′; PFK1-F: 5′-GGCTATGAACTGGATGTC-3′ and PFK1-R: 5′-CCGAATCTGGAGTATTGG-3′; LDHA-F: 5′-GATGATGTCTTCCTTAGTGTT-3′ and LDHA-R: 5′-AGTCAGAGTCACCTTCAC-3′; GLUT1-F: 5′-TATGTGGAGCAACTGTGT-3′ and GLUT1-R: 5′-TGAAGTAGGTGAAGATGAAGA-3′; c-MYC-F: 5′-CCTGGTGCTCCATGAGGAGAC-3′ and c-MYC-R: 5′-CAGACTCTGACCTTTTGCCAGG-3′; 18S-F: 5′-AACTTTCGATGGTAGTCGCCG-3′ and 18S-R: 5′-CCTTGGATGTGGTAGCCGTTT-3′.

### MTT Cell viability assay

HeLa or MDA-MB-231 cells were seeded 2000 per well into 96-well plate. After being cultured for 24 h in a CO_2_ incubator, the cells were treated with DMEM (no glucose), DMEM (no glutamine) or DMEM (no glucose, no glutamine), respectively, for the indicated time. At different time points, 20 *μ*l of MTT (Sigma) solution (5 mg/ml) was added into each well. After incubation for 4 h at 37 °C, the medium was replaced with 150 *μ*l of DMSO, and the plate was allowed to shake on a plate shaker for 15 min. Absorbance at 570 and 650 nm were measured using BioTek Synergy NEO (BioTek, Winooski, VT, USA). Each group was repeated for six times. Values are showed as fold changes *versus* 0-h group.

### Statistical analysis

All the data were shown as mean±S.E.M. Comparison between two groups were performed by two-tailed student’s *t*-test using GraphPad Prism (San Diego, CA, USA).

## Figures and Tables

**Table 1:** Summary of c-MYC protein level changes under GD condition for 12 h as measured by Western blot

*Cell line*	*C-myc change*	*TP53*	*PIK3CA*	*Ras*
293T	Decrease	WT	WT	WT
HeLa	Decrease	WT	WT	WT
MDA-MB-231	Increase	R280K	WT	G13D
MCF7	Decrease	WT	E545K	WT
HCT116	Decrease	WT	H1047R	G13D
HT-29	Increase	R273H	P449T	WT
SW620	Increase	R273H	WT	G12V
Hep3B	Increase	null	WT	WT
H1299	Increase	null	WT	WT
SK-OV-3	Increase	S90fs*33	H1047R	WT
T-24	Decrease	Y126*	WT	WT
K562	Decrease	Q136fs*13	WT	WT
A549	Increase	WT	WT	G12S
BxPC-3	Increase	Y220C	WT	WT
PANC-1	Increase	R273H	WT	G12D
PLC	Increase	R249S	WT	WT
HuH-7	Increase	Y220C	WT	WT

## Abbreviations

GD: glucose deprivation
HIF1: hypoxia-inducible factor 1
GLUT1: glucose transporter 1
PFK: phosphofructokinase
PGK1: phosphoglycerate kinase 1
LDHA: lactate dehydrogenase A
PKM2: M2 isoform of pyruvate kinase M
PFKFB3: phosphofructokinase/fructose-2,6-bisphosphatase B3 gene
HK1: hexokinase 1
GLS: glutaminase
TIGAR: TP53-induced glycolysis and apoptosis regulator
SCO2: synthesis of cytochrome C oxidase 2
CHX: cycloheximide
GSK3: glycogen synthase kinase 3.
